# From anxiety and depression to non-suicidal self-injury in youths: the mediating effect of neuroticism and the moderating effect of thyroid hormones

**DOI:** 10.3389/fpsyt.2026.1857915

**Published:** 2026-07-03

**Authors:** Hui Ma, Jing Zhang, Tiandao Wang, Fang Huang, Lei Li

**Affiliations:** 1 Psychological Counseling and Treatment Center, Hainan Provincial Anning Hospital, Haikou, China; 2 Department of Clinical Laboratory, Hainan Provincial Anning Hospital, Haikou, China; 3 Institute of Mental Health, Hainan Provincial Anning Hospital, Haikou, China

**Keywords:** adolescents, young adults, neuroticism, non-suicidal self-injury, thyroid hormones

## Abstract

**Objective:**

Non-suicidal self-injury (NSSI) is a common but maladaptive behavior among adolescents. Previous studies demonstrated a significant association of anxiety and depression with NSSI. However, the psychological and neuroendocrine mechanisms through which negative emotions influence NSSI remain unclear. The present study aimed to investigate whether the personality trait of neuroticism mediates the relationship between negative emotions and NSSI, and whether thyroid hormones moderate this pathway.

**Methods:**

A total of 104 Han Chinese adolescents and young adults (aged 12–22 years) who exhibited NSSI behaviors were recruited. The participants completed questionnaires to assess NSSI severity (Questionnaire for Middle School Students’ Behavior, QMSSB), depressive symptoms (Hamilton Depression Rating Scale, HAMD), anxiety symptoms (Hamilton Anxiety Rating Scale, HAMA) and personality traits (Eysenck Personality Questionnaire, EPQ). Venous blood was collected for thyroid function tests (TT3, TT4, FT3, FT4 and TSH). Mediation and moderation analyses were conducted using the PROCESS plug-in for SPSS software.

**Results:**

Neuroticism mediated the relationship between emotional symptoms and NSSI. For HAMA in particular, neuroticism fully mediated the association with NSSI (effect = 0.0798, 95% bootstrap confidence interval [0.0387, 0.1367]). TT3 (b = 0.3112, *p* = 0.0147) and FT3 (b = 0.1118, *p* = 0.0371) positively moderated the HAMD-NSSI relationship, while TSH negatively moderated this relationship in the full sample (b = -0.0976, *p* = 0.0011) and remained significant in the high-neuroticism subgroup (β = -1.1116, *p* = 0.0026). Following FDR correction, TT3 (*q* = 0.0245), FT3 (*q* = 0.0412) and TSH (*q* = 0.0055) were found to significantly moderate the HAMD-NSSI relationship in the entire sample. The negative moderating effect of TSH remained significant in the high-neuroticism subgroup (*q* = 0.0078). However, no moderating effects in the low-neuroticism subgroup survived FDR correction (all *q* > 0.05). No significant moderating effects of thyroid hormones were found on the HAMA-NSSI pathway (all *p* > 0.05).

**Conclusions:**

Neuroticism mediates the mood-NSSI link. TT3 and FT3 positively moderate the depression-NSSI link, while TSH negatively moderates it. This negative moderating effect of TSH remains significant only in the high-neuroticism subgroup after FDR correction. These findings integrate psychological and neuroendocrine mechanisms for the identification and intervention of NSSI risk in youths.

## 1 Introduction

Non-suicidal self-injury (NSSI) is defined as deliberately damaging body tissue in a socially unacceptable manner without suicidal intent, resulting in no death (1). Common forms of NSSI include cutting, scratching, hitting, burning, and pulling out hair or skin (2). Reflecting its clinical significance, the fifth edition of the Diagnostic and Statistical Manual of Mental Disorders (DSM-5) includes NSSI in Section III as a condition proposed for further research (1). NSSI behavior typically emerges in early adolescence and is particularly prevalent among adolescents. It is estimated to affect approximately 6-8% of adults (3), 17-18% of adolescents in the general population (4), and up to 40-80% of adolescents in clinical settings (5). Furthermore, the increasing global incidence of NSSI establishes it as a pressing public health concern, as it poses a serious and multifaceted threat to adolescent well-being and development. Therefore, investigating the underlying psychophysiological pathways of NSSI is crucial for developing effective prevention and intervention strategies, which can in turn enhance the mental health of adolescents and young adults and promote their healthy development.

Previous studies have consistently linked NSSI with depressive and anxious moods. Longitudinal studies have shown that depression predicts the continuation of NSSI behaviors in adolescents (6–8). Similar results have been found regarding anxiety and NSSI (9). Accordingly, we hypothesize that depressive and anxious moods are significantly associated with and predictive of NSSI.

Beyond the direct effects of anxious and depressive moods on NSSI, personality traits, particularly neuroticism, may play a significant mediating role. Neuroticism is a stable personality trait characterized by emotional instability, heightened reactivity to negative stimuli and poor impulse control, and is a key dimension of the Eysenck Personality Questionnaire (EPQ) developed by Hans Eysenck (10). Impulsivity, a related construct, has also been associated with NSSI (11), but neuroticism more directly captures the emotional vulnerability that links mood symptoms to self-injury. People who score highly on neuroticism tend to experience more frequent and intense negative emotions, including anxiety and depression, and are less capable of regulating these emotions effectively (10, 12). Importantly, neuroticism has been consistently linked to maladaptive coping behaviors, such as NSSI. Several studies have showed that neuroticism was positively associated with self-harm, and was an independent predictor of self-harm risk (13, 14).

In addition to neuroticism, the EPQ also measures two other key personality dimensions: psychoticism and extraversion (10). Psychoticism is characterized by aggressiveness, interpersonal coldness and egocentricity, whereas extraversion reflects sociability and positive emotionality. Evidence linking these two dimensions to NSSI and emotional symptoms is less consistent than for neuroticism. Some studies have reported higher psychoticism in adolescents with NSSI (15, 16) and positive correlations between psychoticism and anxiety/depression (17). Regarding extraversion, some studies have observed lower extraversion in individuals with anxiety or depression (18, 19), whereas the association between extraversion and NSSI remains unclear (16). Given the inconsistent evidence and exploratory nature of psychoticism and extraversion, the present study focuses its main hypothesis on neuroticism as the proposed mediator between mood symptoms and NSSI.

Beyond psychological mechanisms, neuroendocrine function may also play a regulatory role in the link between negative moods and NSSI. Thyroid hormones play a crucial role in regulating mood (20), controlling impulse (21) and responding to stress responsiveness (22), all of which are relevant to vulnerability to NSSI. Five serum parameters are routinely measured to assess thyroid function: total triiodothyronine (TT3), total thyroxine (TT4), free triiodothyronine (FT3), free thyroxine (FT4) and thyroid-stimulating hormone (TSH). A recent meta-analysis found that serum TT3 and FT3 levels decreased significantly in patients experiencing depressive episodes of bipolar disorder, compared with healthy controls (23). Another study demonstrated that adolescents with NSSI exhibited lower FT3/FT4 ratio values than healthy controls, and there were negative correlations between FT3, the FT3/FT4 ratio and depression severity (24). TSH has been demonstrated to be negatively associated with NSSI in male adolescents with depression (25). However, to date, no study has systematically examined whether thyroid hormones moderate the pathway from negative moods to NSSI, or whether such moderation depends on personality traits such as neuroticism. As neuroticism is characterized by heightened emotional reactivity and impaired inhibitory control, individuals with high level of neuroticism may be particularly susceptible to neuroendocrine variations that facilitate or constrain the translation of depressive symptoms into self-injurious behaviors.

Beyond the mediating role of neuroticism, individual differences in neuroticism may also modulate the effect of thyroid hormones on the depression-NSSI pathway. Individuals with high neuroticism may be more susceptible to neuroendocrine variations due to their heightened emotional reactivity and impaired inhibitory control (10, 12), whereas those with low neuroticism may have a lower baseline risk of NSSI (14), leaving less room for further biological modulation. Therefore, we examined whether the moderating effect of TSH on the depression-NSSI relationship is conditional on neuroticism level.

To summarize, the aim of this study is to integrate psychological and neuroendocrine perspectives by testing a moderated mediation model. This model examines: (1) whether neuroticism (as a trait index of impulsivity) mediates the relationship between negative moods (anxiety/depression) and NSSI; (2) whether thyroid hormone levels moderate this pathway; and (3) whether the moderating effect of TSH on the depression-NSSI relationship is conditional on neuroticism level. Clarifying these conditional mechanisms could help to identify at-risk adolescents and young adults earlier and inform personalized interventions that target both psychological vulnerability and neuroendocrine markers.

## 2 Materials and methods

### 2.1 Participants

Adolescents and young adults with NSSI were recruited from a psychiatric hospital in Hainan Province, China. The inclusion criteria were (1) Outpatients or inpatients presenting for their first visit to this hospital who have been diagnosed with NSSI disorder by two senior psychiatrists based on DSM-5 criteria, and who are treatment-naïve (have not received any prior pharmacological or psychological treatment for NSSI); (2) Aged 12−22 years; (3) Of Han ethnicity. Exclusion criteria were (1) Patients with organic disorders of the central nervous system; (2) Patients with unresolved chronic or severe physical illnesses; (3) Patients with comorbid neurodevelopmental disorders, schizophrenia or other primary psychotic disorders. Written informed consent was obtained from all the participants. For participants under the age of 18, additional written informed consent was obtained from their legal guardians. Upon enrollment, each participant completed several psychological assessment questionnaires, including the Questionnaire for Middle School Students’ Behavior (QMSSB), the Hamilton Depression Scale (HAMD), the Hamilton Anxiety Scale (HAMA), and the EPQ. A 4 mL sample of venous blood was obtained from each participant for thyroid function testing.

A total of 104 Han Chinese participants were recruited. They were aged between 12 and 22 years. The mean age was 16.16 years and the standard deviation (SD) was 2.51 years. The study sample included 17 males (16.35%) and 87 females (83.65%). All the protocols in the present study were approved by the Ethics Committee of Hainan Provincial Anning Hospital (Approval No. 2024-003-01) in Haikou, China.

### 2.2 Methods

#### 2.2.1 Questionnaires

The Chinese Questionnaire for Middle School Students’ Behavior (QMSSB), which was developed by Ying Zheng (26) based on the Functional Assessment of Self-Mutilation (FASM) created by Lloyd et al. (27), was used to collect the general demographic information and assess the NSSI behaviors over the past year. The QMSSB comprises three sections. Section 1 primarily consists of demographic variables including the gender, age, grade level, ethnicity, place of origin, parental occupation type and monthly household income. Section 2 lists 20 distinct NSSI behaviors and how frequently they occur, scored on a four-point scale ranging from 0 to 3. Section 3 enumerates 38 reasons or purposes for NSSI behaviors, also scored on a four-point scale ranging from 0 to 3. The present study primarily uses Sections 1 and 2 of the QMSSB. The scores from all items of Section 2 contribute to a total score reflecting the overall severity of NSSI. The section 2 of the QMSSB has demonstrated good reliability in Chinese adolescents: the one-month test-retest reliability coefficients were 0.79 (26).

The 24-item Chinese version of the Hamilton Depression Scale (HAMD), originally developed by Hamilton (28), is a clinician-administered scale for assessing the severity of depressive symptoms. It is usually carried out through a clinical interview, with the assessment period covering the past week. Most projects use a five-point scale ranging from 0 to 4 points, while a few use a three-point scale ranging from 0 to 2 points. The HAMD measures seven dimensions of depression, including anxiety/somatization, weight, cognitive disturbance, diurnal variation, retardation, sleep disturbance and hopelessness. Scores from all items are summed to produce a total score, with higher scores indicating greater severity of depression. The HAMD has demonstrated excellent reliability in Chinese populations. Inter-rater reliability ranges from 0.88 to 0.99 in Chinese validation studies (Shanghai Mental Health Center; 14-unit national collaborative study) (29).

The 14-item Chinese version of the Hamilton Anxiety Scale (HAMA), originally developed by Hamilton (30), is a clinician-administered scale for assessing the severity of anxious symptoms. It is usually carried out through a clinical interview, with the assessment period covering the past week. Each item is scored on a 0-4 scale. The HAMA is structured to evaluate two broad symptom groups: psychic anxiety (e.g., anxious mood, tension, fears and difficulty concentrating) and somatic anxiety (e.g., muscular, sensory, cardiovascular, respiratory and gastrointestinal symptoms). The total score reflects overall anxiety severity and is obtained by summing the scores for all items. The HAMA has demonstrated excellent reliability in Chinese populations. Inter-rater reliability for the total score was 0.93 after more than 10 systematic training sessions (Shanghai Mental Health Center), with individual item coefficients ranging from 0.83 to 1.00 (29).

The Chinese version of the EPQ, which was originally developed by Hans Eysenck and his wife, was used to measure core personality traits. Two versions were used in the present study: the Adolescent Version (for ages 7-15) and the Adult Version (for ages 16 and over). Each EPQ version includes 88 items and consists of a series of questions that are answered using a yes/no scale. The EPQ is structured around several clearly defined, theoretically grounded personality dimensions, most commonly including Psychoticism (P), Extraversion (E) and Neuroticism (N), as well as a Lie Scale (L) to assess the validity of responses. Scores on the P, E and N scales are calculated separately to provide a personality profile. The Chinese version of the EPQ has demonstrated good reliability. For the adult version, split-half reliability ranges from 0.65 to 0.88, and test-retest reliability (one-month interval) ranges from 0.67 to 0.92. For the youth version, split-half reliability ranges from 0.65 to 0.88, and test-retest reliability (one-month interval) ranges from 0.65 to 0.86 (31).

All participants were asked to complete every item in each of the four questionnaires.

#### 2.2.2 Thyroid function testing

Thyroid function was assessed using five serum parameters routinely measured to comprehensively reflect activity of the hypothalamic–pituitary–thyroid (HPT) axis: TT3, TT4, FT3, FT4 and TSH. To control for the known diurnal rhythm of TSH, all blood samples were collected in the early morning (between 6:00 and 6:30 AM) after an overnight fast. Following collection, peripheral blood samples were immediately transferred to the department of clinical laboratory in the hospital, where the concentrations of these five parameters were determined using a chemiluminescent immunoassay (CLIA). Blood samples were collected on the same day as the psychological assessments. All assay results were promptly provided to the investigator for recording.

### 2.3 Statistical analysis

Statistical analyses were performed using SPSS version 26.0 (SPSS Inc., Chicago, Illinois, USA) and the PROCESS version 3.2 plug-in. Data are presented as the mean ± standard deviation (SD), or as a frequency and percentage. The analysis proceeded in a sequential and conditional manner.

First, Pearson correlation analysis was conducted to examine bivariate associations among all the study variables.

Second, a mediation analysis was performed using PROCESS Model 4 to test whether neuroticism mediated the relationship between emotional symptoms (HAMD and HAMA) and NSSI severity (QMSSB). The significance of the indirect effects was examined using bootstrapping with 5,000 resamples. Bias-corrected 95% confidence intervals (CIs) that did not include zero were considered statistically significant.

Third, if a significant mediation effect was detected, moderation analyses were conducted using PROCESS Model 1 to examine the following: (a) whether the five thyroid function parameters moderated the direct relationships between emotional symptoms and NSSI; and (b) whether these thyroid parameters moderated the individual paths of the mediation model, i.e., the path from emotional symptoms to neuroticism, and the path from neuroticism to NSSI. All continuous variables were mean-centered prior to moderation analysis. A moderation effect was considered significant if the *p*-value for the interaction term was less than 0.05. When a significant interaction was detected, simple slope tests and effect plots were used to investigate the direction and pattern of the moderation effect.

Fourth, if any significant moderation effects were identified, subgroup analyses would be conducted and stratified by neuroticism level, based on a median split of EPQ neuroticism scores.

An uncorrected *p* value of less than 0.05 was considered statistically significant unless otherwise specified. To control for Type I error inflation due to the number of statistical tests performed, the false discovery rate (FDR) method (32) was pre-specified as the correction procedure for all moderation analyses. FDR correction would be applied to the original *p*-values of moderation effects that reached nominal significance (*p* < 0.05). This correction was planned for: (a) the five moderation tests on the HAMD-NSSI relationship in the full sample; (b) the five moderation tests on the HAMA-NSSI relationship in the full sample; and (c) the moderation tests conducted within the high- and low-neuroticism subgroups. Following the Benjamini-Hochberg procedure, FDR-adjusted *p* (*q*) values of less than 0.05 were considered statistically significant, where significance is determined by comparing the original *p*-value to the critical value (i/m) × 0.05.

## 3 Results

### 3.1 Correlation analyses

The main variables studied were emotional symptoms (HAMD and HAMA), NSSI severity (QMSSB), personality traits (EPQ) and thyroid function parameters (TT3, TT4, FT3, FT4 and TSH). Descriptive statistics for all study variables are presented in **Table 1**, and Pearson correlation coefficients among them are shown in **Table 2**. As shown, NSSI severity (QMSSB) was significantly positively correlated with both HAMD (r = 0.468, *p* < 0.01) and HAMA (r = 0.267, *p* < 0.01), as well as with neuroticism (r = 0.493, *p* < 0.01). Neuroticism itself was also significantly positively correlated with HAMD (r = 0.360, *p* < 0.01) and HAMA (r = 0.309, *p* < 0.01). For other personality traits, psychoticism showed significant positive correlations with HAMA (r = 0.344, *p* < 0.01) and extraversion (r = 0.203, *p* < 0.05). For thyroid function parameters, TSH showed a significant negative correlation with TT4 (r = -0.237, *p* < 0.05) and FT4 (r = -0.225, *p* < 0.05).

**Table 1:** Descriptive statistics of the study variables.

Study variables	Mean	Standard deviation	Unit
HAMD	31.93	9.85	–
HAMA	23.50	9.01	–
P	60.53	14.23	–
E	37.16	11.72	–
N	70.05	7.49	–
QMSSB	26.10	5.13	–
TT3	1.68	0.39	nmol/L
TT4	82.98	18.36	nmol/L
FT3	4.62	0.84	pmol/L
FT4	15.83	2.97	pmol/L
TSH	2.01	1.67	μIU/ml

HAMD, Hamilton Depression Rating Scale; HAMA, Hamilton Anxiety Rating Scale; P, Psychoticism; E, Extraversion; N, Neuroticism; QMSSB, Questionnaire for Middle School Students’ Behavior; TT3, total triiodothyronine; TT4, total thyroxine; FT3, free triiodothyronine; FT4, free thyroxine; TSH, thyroid-stimulating hormone. All questionnaire scores are presented as total scores without units.

**Table 2:** Pearson correlations among emotional symptoms, personality traits, thyroid function parameters and self-injury severity (r).

Study	Emotional symptoms		Eysenck personality questionnaire			Thyroid function parameters					Self-injury severity
Variables	HAMD	HAMA	P	E	N	TT3	TT4	FT3	FT4	TSH	QMSSB
HAMD	1										
HAMA	0.435^**^	1									
P	0.031	0.344^**^	1								
E	-0.044	-0.143	0.203^*^	1							
N	0.360^**^	0.309^**^	-0.028	-0.095	1						
TT3	0.126	-0.021	0.021	-0.036	0.005	1					
TT4	0.121	-0.036	0.026	-0.101	-0.029	0.538^**^	1				
FT3	0.003	-0.170	-0.055	-0.065	-0.003	0.814^**^	0.410^**^	1			
FT4	0.102	-0.113	-0.117	-0.172	0.014	0.234^*^	0.803^**^	0.318^**^	1		
TSH	-0.067	0.015	0.038	0.129	0.173	-0.089	-0.237^*^	-0.050	-0.225^*^	1	
QMSSB	0.468^**^	0.267^**^	0.003	-0.020	0.493^**^	0.004	0.016	-0.060	0.039	0.016	1

HAMD, Hamilton Depression Rating Scale; HAMA, Hamilton Anxiety Rating Scale; P, Psychoticism; E, Extraversion; N, Neuroticism; TT3, Total Triiodothyronine; TT4, Total Thyroxine; FT3, Free Triiodothyronine; FT4, Free Thyroxine; TSH, Thyroid-Stimulating Hormone; QMSSB, Questionnaire for Middle School Students’ Behavior.

*p < 0.05, **p < 0.01.

### 3.2 Analysis of the mediating effect of personality traits on the relationship between anxiety/depression symptoms and self-injury severity

**Figure 1:**
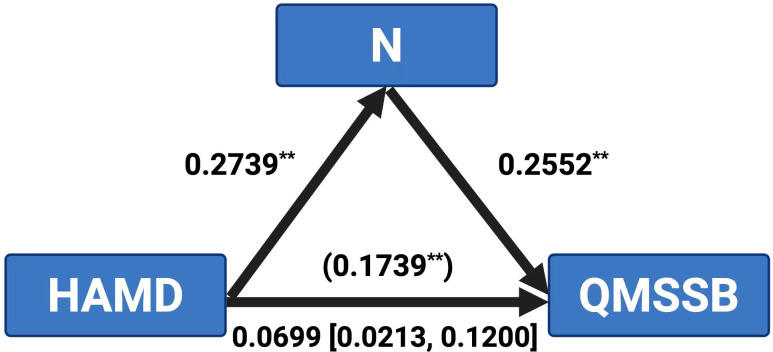
Mediation model examining the indirect relationship between HAMD and QMSSB. Indirect effect(s) of HAMD on QMSSB: 0.0699 [0.0213, 0.1200]. Direct effect(s) of HAMD on QMSSB: 0.1739. **p < 0.01.

**Figure 2:**
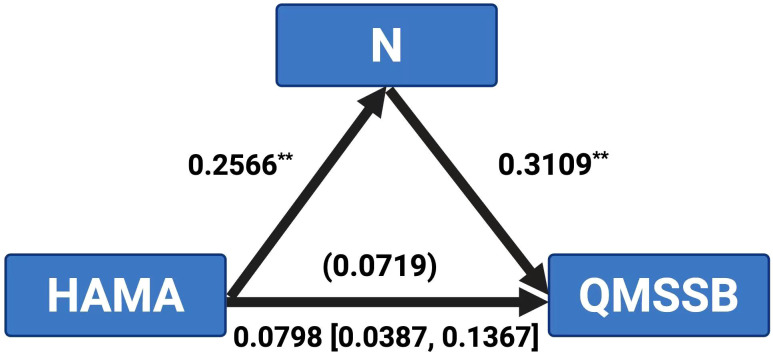
Mediation model examining the indirect relationship between HAMA and QMSSB. Indirect effect(s) of HAMA on QMSSB: 0.0798 [0.0387, 0.1367]. Direct effect(s) of HAMA on QMSSB: 0.0719. **p < 0.01.

Neuroticism was demonstrated to mediate the relationship between each emotional−symptom variable (HAMA and HAMD) and self−injury severity (QMSSB). Results are summarized in **Table 3** and illustrated in **Figures 1**, **2**. Neuroticism served as a significant mediator in both models. The paths from each emotional symptom to neuroticism (path a) and from neuroticism to QMSSB (path b) were all statistically significant (*p* < 0.01). For HAMD, the path coefficients were a = 0.2739 (*p* < 0.01) and b = 0.2552 (*p* < 0.01), and for HAMA, a = 0.2566 (*p* < 0.01) and b = 0.3109 (*p* < 0.01).

**Table 3:** Results of mediation analyses with neuroticism as a mediator.

Model (X→M→Y)	Path a (X→M)	Path b (M→Y)	Direct effect	Indirect effect (a×b) [95% Boot CI]	Mediation type
HAMD→N→QMSSB	0.2739**	0.2552**	0.1739**	0.0699 [0.0213, 0.1200]	Partial
HAMA→N→QMSSB	0.2566**	0.3109**	0.0719	0.0798 [0.0387, 0.1367]	Complete

X = Independent Variable; M = Mediator (Neuroticism); Y = Dependent Variable (QMSSB); N = neuroticism. Path coefficients are unstandardized. Bootstrap confidence intervals (Boot CI) that do not include zero indicate a significant indirect effect.

*p < 0.05, **p < 0.01.

For the relationship between HAMA and QMSSB, the direct effect was not significant (β = 0.0719, *p* = 0.1641), whereas the indirect effect via neuroticism was significant (effect = 0.0798, 95% Boot CI [0.0387, 0.1367]). This suggests that neuroticism completely mediates the association between anxiety and self−injury, as HAMA’s impact on QMSSB is entirely due to its ability to increase neuroticism.

By contrast, both the direct and indirect effects of HAMD were significant. The direct effect was 0.1739 (*p*< 0.01) and the indirect effect was 0.0699 (95% Boot CI [0.0213, 0.1200]). These results suggest that neuroticism plays a partial mediating role in the relationship between depression and self−injury. HAMD affects QMSSB both directly and indirectly by increasing neuroticism. The proportion of the total effect mediated by neuroticism was 28.67% (0.0699/0.2438).

Psychoticism and extraversion were not further examined as mediators, given their lack of significant bivariate associations with QMSSB (see **Table 2**). The mediating role of neuroticism is summarized in **Table 3**.

### 3.3 Analysis of the moderation effect of thyroid function on the relationship between anxiety/depression symptoms and self-injury severity

Of the five analyzed thyroid function indicators, TT3, FT3 and TSH demonstrated significant moderating roles in the relationship between HAMD and QMSSB, though in distinct patterns. No significant moderating effects were observed for TT4 (b = 0.0025, *p* = 0.3281) or FT4 (b = 0.0040, *p* = 0.7933) on the relationship between HAMD and QMSSB.

**Figure 3:**
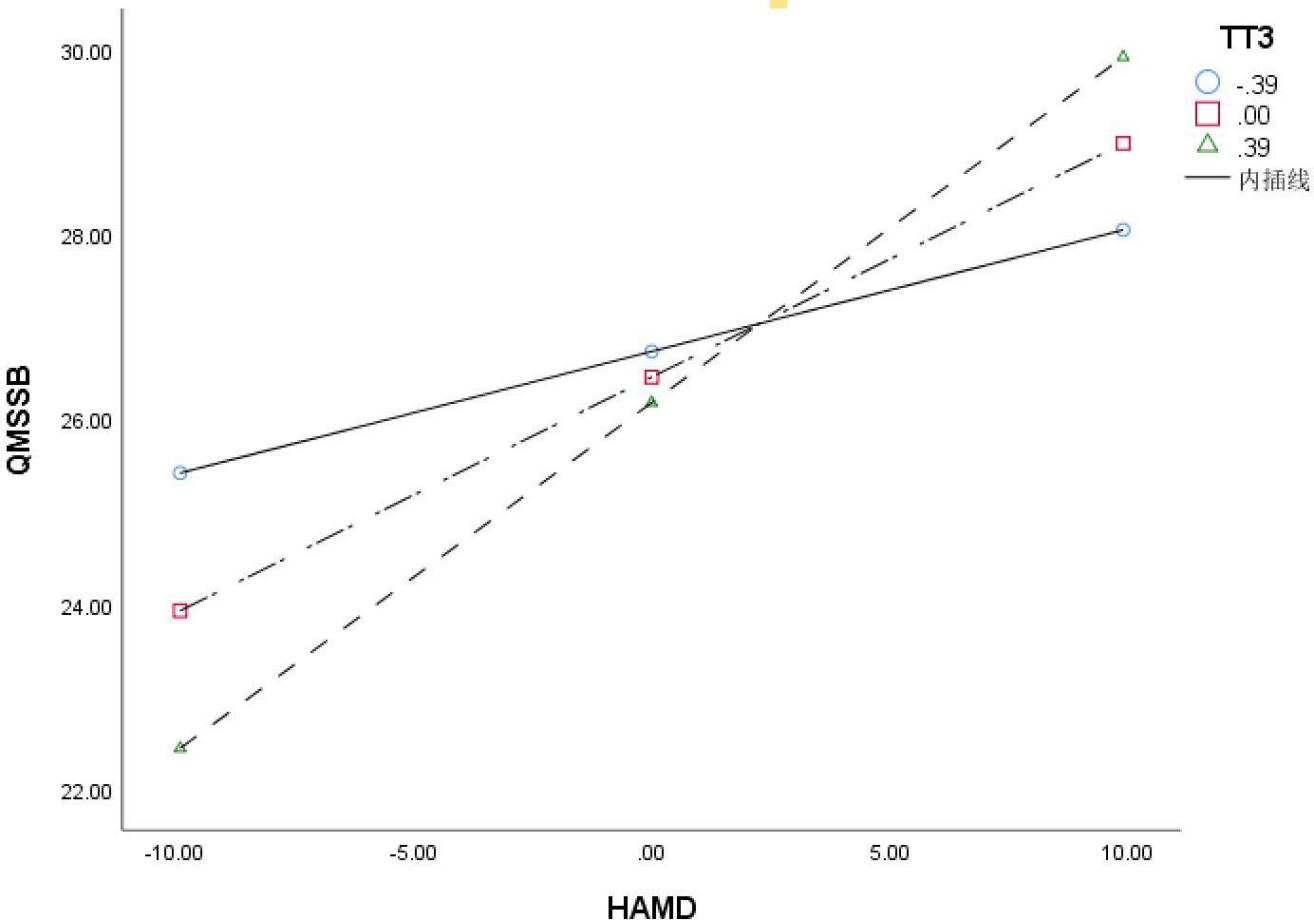
Moderating effect of different TT3 levels on the relationship between depression (HAMD) and self-injury severity (QMSSB). Simple slopes are plotted at low (−1 SD), mean, and high (+1 SD) levels of TT3. The interaction was significant (b = 0.3112, *p* = 0.0147).

The interaction between HAMD and TT3 was significant (b = 0.3112, *p* = 0.0147). As **Figure 3** showed, according to simple slope analysis, the unstandardized coefficient of HAMD on QMSSB increased with higher levels of TT3: at low level of TT3 (−1 SD), the effect was 0.1329 (*p* = 0.0466); at the mean level of TT3, the effect was 0.2554 (*p* < 0.001); and at high level of TT3 (+1 SD), the effect increased to 0.3779 (*p* < 0.001).

**Figure 4:**
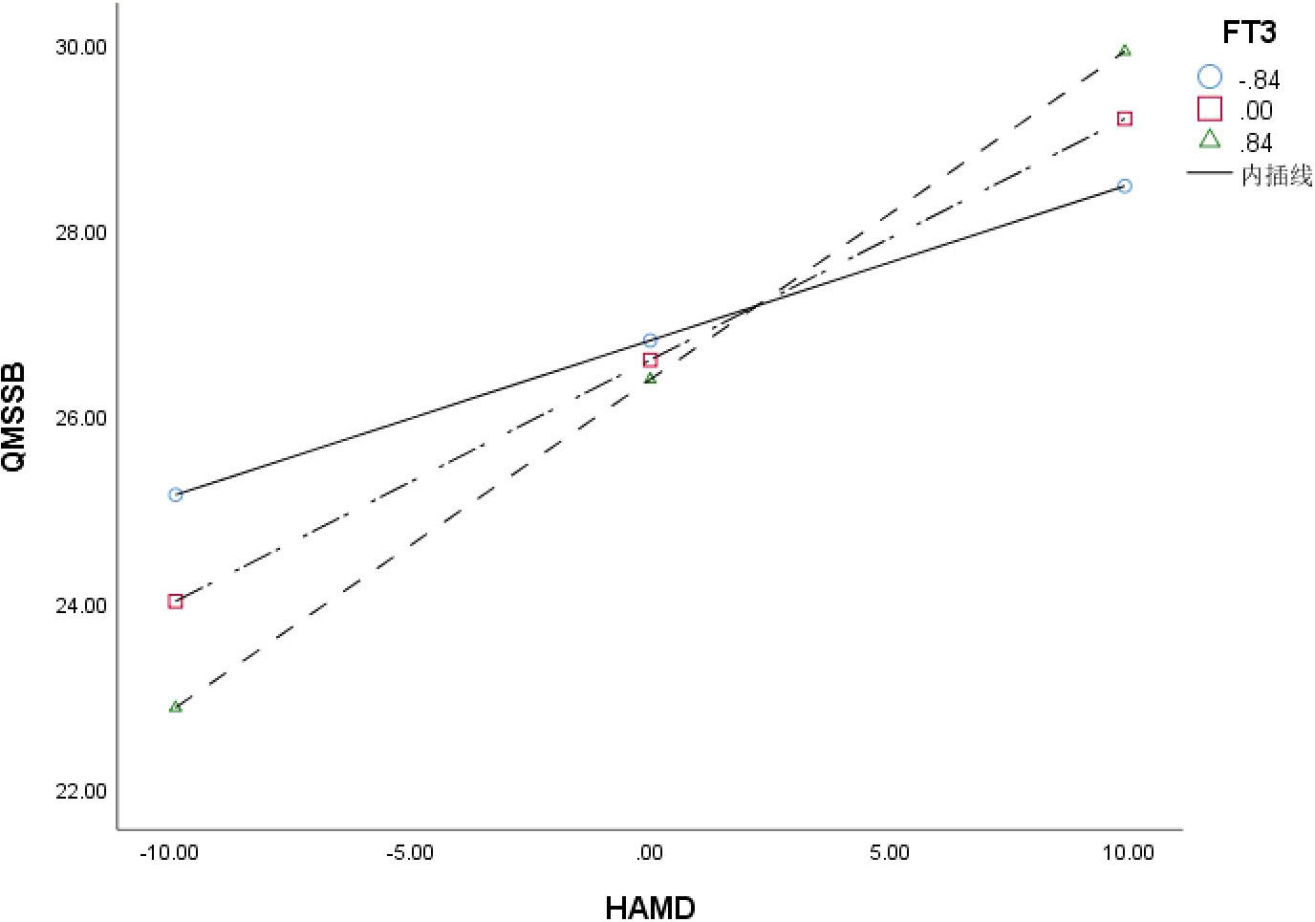
Moderating effect of different FT3 levels on the relationship between depression (HAMD) and self-injury severity (QMSSB). Simple slopes are plotted at low (−1 SD), mean, and high (+1 SD) levels of FT3. The interaction was significant (b = 0.1118, *p* = 0.0371).

Similarly, the interaction between HAMD and FT3 was significant (b = 0.1118, *p* = 0.0371). Higher levels of FT3 strengthened this relationship. The effect of HAMD on QMSSB was 0.1677 (*p* = 0.0054) at low FT3 level, 0.2621 (*p* < 0.001) at the mean level, and 0.3565 (*p* < 0.001) at high FT3 level (see **Figure 4**).

**Figure 5:**
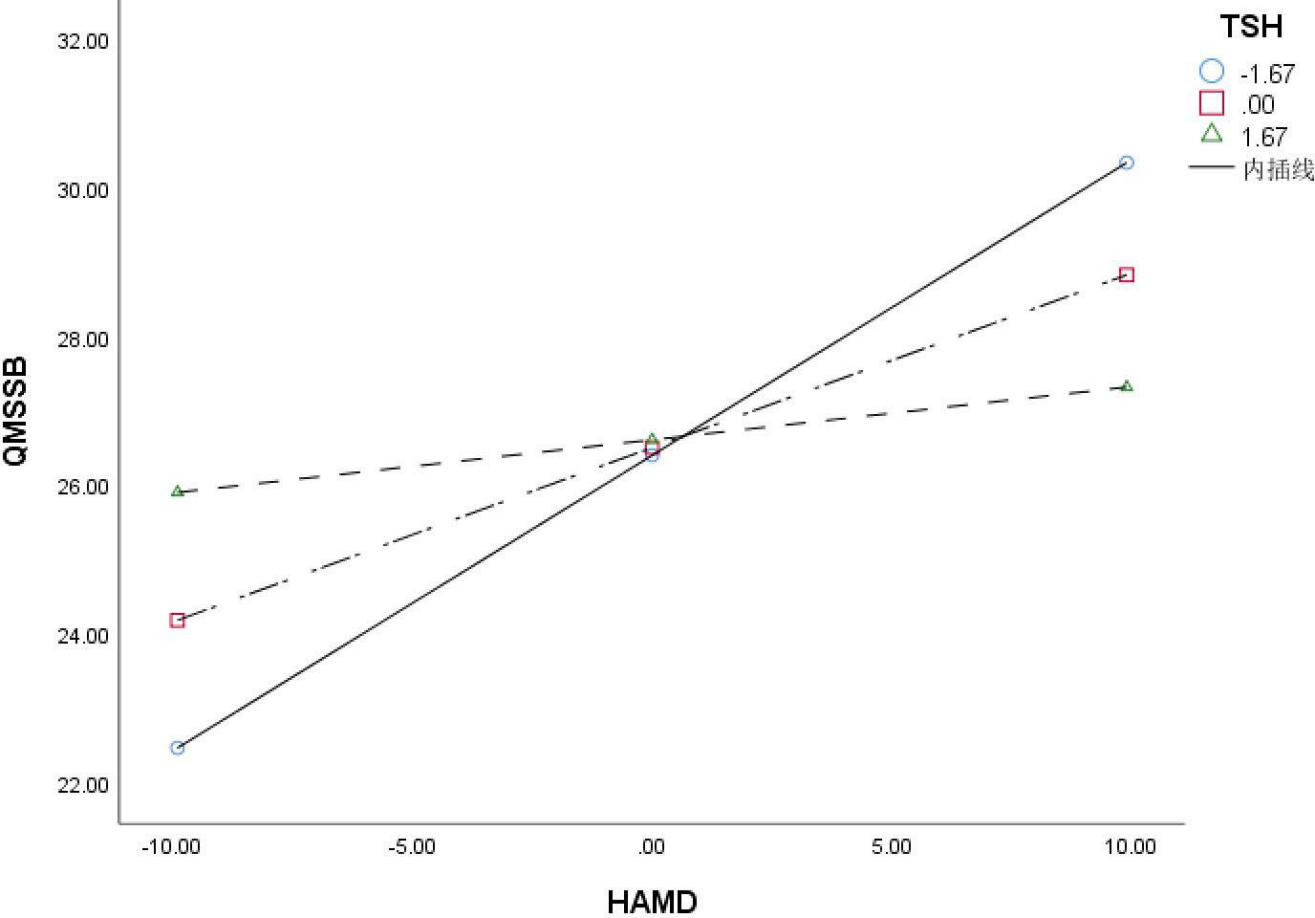
Moderating effect of different TSH levels on the relationship between depression (HAMD) and self-injury severity (QMSSB). Simple slopes are plotted at low (−1 SD), mean, and high (+1 SD) levels of TSH. The interaction was significant (b = −0.0976, *p* = 0.0011).

In contrast, the interaction between HAMD and TSH was also significant (b = −0.0976, *p* = 0.0011), but in the opposite direction. Higher TSH levels attenuated the effect of HAMD on QMSSB. At low TSH level (−1 SD), the effect was 0.3975 (*p* < 0.001); at the mean TSH level, it was 0.2345 (*p* < 0.001); and at high TSH level (+1 SD), the effect decreased to 0.0715 (*p* = 0.2961), becoming non−significant (see **Figure 5**).

The FDR correction was applied to the five moderation tests: HAMD × TT3, FT3, TT4, FT4 and TSH. Following this correction, the moderating effects of TT3 (*q* = 0.0245), FT3 (*q* = 0.0412) and TSH (*q* = 0.0055) remained significant.

Furthermore, no significant moderating effects were observed for any of the five thyroid function indicators in the relationship between HAMA and QMSSB (all *p*>0.05). Additionally, none of the EPQ personality traits (Psychoticism, Extraversion or Neuroticism) significantly moderated the relationship between HAMA/HAMD and QMSSB (all *p* > 0.05).

### 3.4 Analysis of the moderation effect of thyroid function on the relationship between anxiety/depression symptoms and neuroticism, and between neuroticism and self-injury severity

No significant moderating effects were observed for any of the five thyroid function indicators in the relationships between HAMD/HAMA symptoms and neuroticism, or between neuroticism and QMSSB (all *p* > 0.05). These null findings indicate that thyroid hormones do not exert their moderating effects through the specific pathways of the mediation model.

### 3.5 Analysis of the moderation effect of thyroid function in the relationship between depression symptom and self-injury severity: subgroup analyses by neuroticism level

Given the significant moderating effects of TSH on the direct depression-NSSI relationship found in the full sample (Section 3.3) and consistent with our hypothesis that this moderating effect may be conditional on neuroticism level, we next examined whether this moderating effect varied by individual differences in levels of neuroticism. Participants were divided into high (n = 61) and low (n = 43) neuroticism subgroups based on the median EPQ neuroticism score (70.05). Moderator analyses were conducted separately for each subgroup, with HAMD as the independent variable, QMSSB as the dependent variable, and the three thyroid parameters that showed significant moderating effects in the full sample (TT3, FT3 and TSH) as moderators.

In the high neuroticism subgroup, TSH was found to significantly moderate the relationship between HAMD and QMSSB (β = -1.1116, SE = 0.0346, t = -3.22, *p* = 0.0026). The negative coefficient indicated that higher TSH levels reduced the positive effect of HAMD on QMSSB. Simple slope analysis showed that, at high TSH levels (+1 SD), the effect of HAMD on QMSSB was substantially reduced; conversely, at low TSH levels (-1 SD), the effect was stronger.

TT3 showed a trend-level positive moderating effect (β = 0.3873, SE = 0.1995, t = 1.94, *p* = 0.0598), suggesting that higher TT3 levels strengthened the depression-NSSI link, although this effect did not reach conventional statistical significance. FT3 did not show a significant moderating effect (β = 0.0980, SE = 0.0824, t = 1.19, *p* = 0.2418).

Following FDR correction for the three tests in the high-neuroticism subgroup, TSH remained significant (*q* = 0.0078), whereas TT3 and FT3 did not reach significance (both *q* > 0.05).

In the low neuroticism subgroup, TSH showed a negative moderating effect (β = -0.1016, SE = 0.0473, t = -2.15, *p* = 0.0365), TT3 showed a trend-level positive moderation (β = 0.2650, SE = 0.1374, t = 1.93, *p* = 0.0593), and FT3 did not demonstrate a significant effect (β = 0.0766, SE = 0.0603, t = 1.27, *p* = 0.2094).

Following FDR correction for the three tests in the low-neuroticism subgroup, none of these effects remained significant (all *q* > 0.05).

## Discussion

In summary, the present study has demonstrated that neuroticism is a robust mechanism linking various forms of emotional distress to self-injury behaviors. This role is particularly evident in clinician-rated anxiety (HAMA), where neuroticism acts as a complete mediator. This suggests that the impact of anxiety on self-injury behaviors depends entirely on the individual level of neuroticism. For clinician-rated depression (HAMD), however, neuroticism is an important but not exclusive pathway.

Previous studies have established independent associations between the personality traits of neuroticism or impulsivity and negative emotions (33), as well as between these personality traits and self-injury behaviors (11). Furthermore, Liao et al.’s study also demonstrated that neuroticism significantly positively predicted depression and NSSI (14). Our study provides novel empirical evidence for the mediating role of neuroticism in the pathway linking clinician-rated anxiety and depression to NSSI severity in youths, which has not been previously demonstrated. This provides a novel mechanism through which to understand that neuroticism is not merely a correlate of anxiety and depression, but a central explanatory pathway through which they translate into self-injury behaviors.

One plausible mechanism is that neuroticism may amplify the perceived intensity of negative emotions, thereby impairing cognitive function and the ability to regulate emotions effectively. This can increase the urge to engage in maladaptive coping behaviors such as self-injury (14, 34). In line with this perspective, recent functional near-infrared spectroscopy (fNIRS) studies have revealed abnormalities in the prefrontal cortex of adolescents with NSSI, such as reduced activation in the prefrontal and temporal lobes (35) and altered hemodynamics in the dorsolateral prefrontal cortex (36). However, findings have not been entirely consistent (37). Building on these findings, our recent fNIRS research has identified prefrontal abnormalities across three groups: youths with NSSI and depression, youths with NSSI but no depression, and healthy controls (unpublished data). This provides further indirect neural evidence of impaired inhibitory control in NSSI behavior.

Interestingly, the mediating role of neuroticism on NSSI differed between anxiety and depression. It fully mediated the anxiety-NSSI relationship, but only partially mediated the depression-NSSI relationship. These differential mediation patterns suggest that anxiety symptoms primarily lead to NSSI through personality vulnerability (i.e., neuroticism), whereas depression influences NSSI via both neuroticism-dependent and neuroticism-independent pathways. The latter may involve other psychological constructs (e.g., hopelessness) (8) or biological regulators (e.g., thyroid hormones) (24, 25, 38). We therefore tested whether thyroid hormones moderate the depression-NSSI pathway.

The findings reveal a significant moderating effect of thyroid function (specifically TT3, FT3 and TSH levels) on the relationship between clinician-rated depression (HAMD) and self-injury severity. Previous studies have established some independent associations between thyroid function and negative emotional states (23) and between thyroid function and self-injury behaviors (24, 38). To our knowledge, this is the first study to demonstrate the moderating role of thyroid function in the pathway linking clinician-rated depression to NSSI severity, rather than merely examining group differences in hormone levels (24, 38).

Specifically, the findings reveal that higher levels of TT3 and FT3 are associated with a stronger positive association between depression and NSSI, whereas higher levels of TSH are associated with a weaker relationship. These opposing moderating patterns suggest that behavioral translation of depressive symptoms may be influenced by different dimensions of thyroid function: peripheral hormone levels (TT3/FT3) versus pituitary feedback (TSH). The distinct moderating roles of TT3/FT3 and TSH can be understood through their neurobiological actions. Thyroid hormones directly regulate cerebral metabolism, neurotransmitter synthesis (particularly serotonin and norepinephrine) and limbic system activity, all of which are critical for mood regulation and impulse control (39–41). Elevated TT3 and FT3 levels may increase neural excitability (39), thereby lowering the threshold for impulsive or self-injurious behaviors during depressive episodes. In contrast, elevated TSH levels may reflect a compensatory feedback mechanism in response to central hypothyroidism or altered set-point regulation of the HPT axis (42). This could dampen emotional reactivity and enhance inhibitory control. For instance, loss of TSHR function *TSHR* knockout mice leads to a blunted TSH signaling response and has been observed to exhibit increased impulsiveness and aggression (43), suggesting that intact TSH signaling may be important for behavioral inhibition. These opposing effects (TSH as a protective factor and TT3 and FT3 as risk factors) highlights the intricate, non-linear nature of the HPT axis’ involvement in stress-related behaviors. Nevertheless, the enhancing effects of TT3 and FT3 did not survive FDR correction in subgroup analyses, warranting cautious interpretation.

Notably, no significant moderating effects of thyroid hormones were observed in relation to the anxiety-NSSI pathway. This negative finding may reflect the distinct neurobiological underpinnings of depression and anxiety. Depression is more closely linked to HPT axis dysfunction (44), whereas anxiety is more closely associated with hypothalamic-pituitary-adrenal (HPA) axis and noradrenergic system dysregulation (45, 46). Consistent with this view, the complete mediation of the anxiety-NSSI relationship by neuroticism suggests that anxiety primarily influences NSSI through personality-based rather than HPT-axis-related neuroendocrine pathways. It should be noted that the potential role of HPA axis measures (e.g., cortisol) was not examined in the present study.

Importantly, the moderating effect of TSH was significant in the full sample and remained significant in the high-neuroticism subgroup after FDR correction, but not in the low-neuroticism subgroup. This pattern suggests that thyroid hormones and neuroticism influence the depression-NSSI pathway through different yet interacting mechanisms. Neuroticism acts as a psychological mediator, explaining how negative emotions lead to NSSI (14, 33, 34), whereas thyroid hormones act as a neuroendocrine moderator, altering the strength of the direct link between depression and NSSI (24, 25, 38). Notably, these two mechanisms do not operate through the same pathway. Thyroid hormones did not significantly moderate the relationships between depression and neuroticism, or between neuroticism and NSSI. Instead, they moderated the direct relationship between depression and NSSI. Therefore, while neuroticism mediates the translation of depression into NSSI, thyroid hormones, particularly TSH, moderate the residual direct effect of depression on NSSI severity in a neuroticism-dependent manner. The protective effect of TSH is enhanced by high levels of neuroticism. This suggests that individuals with heightened emotional reactivity and impaired impulse control may be particularly susceptible to neuroendocrine variations that prevent depressive symptoms from manifesting as behaviors. Among those with a low level of neuroticism, the baseline risk of NSSI may already be low, leaving less scope for further biological modulation. Taken together, these findings suggest that thyroid function acts as a neuroticism-dependent moderator of the depression-NSSI pathway.

### Limitations

The present results should be interpreted in light of certain limitations. First, the sample was exclusively recruited from the Han Chinese population, meaning the results may not be directly applicable to other ethnic groups.

Second, while the subgroup sample sizes (low neuroticism: n = 43; high neuroticism: n = 61) provided adequate power to detect moderate-to-large effects, they may have been insufficient for small effects. The trend-level findings for TT3 in both subgroups (*p* = 0.0598 and 0.0593, respectively) should be interpreted with caution and require replication in larger samples.

Third, the assessment of NSSI severity relied on a self-report questionnaire (QMSSB), which is subject to recall bias. However, the HAMD and HAMA were clinician-administered scales, which minimized potential bias in the assessment of depressive and anxiety symptoms.

Fourth, the cross-sectional design limits causal inferences about the observed associations. Although our mediation and moderation models were theory-driven, longitudinal studies with repeated measurements are needed to establish temporal precedence among negative moods, neuroticism, thyroid function and NSSI.

Fifth, due to the study design, individual item-level data were not retained. Ttherefore, internal consistency reliability (Cronbach’s α) could not be calculated directly from the present sample. Instead, we have reported other types of reliability evidence for these instruments, including inter-rater reliability (for HAMD and HAMA), split-half and test-retest reliability (for EPQ), and test-retest reliability (for QMSSB Section 2), which are available in Chinese validation studies.

Sixth, the study sample was predominantly female (83.65%), reflecting the natural sex distribution of NSSI in clinical settings, where female adolescents are overrepresented (47). While this is consistent with clinical reality, the severe gender imbalance limits the generalizability of our findings. The prevalence, correlates and underlying mechanisms of NSSI may differ between males and females. It is possible that the mediating role of neuroticism or the moderating role of thyroid hormones could operate differently in male adolescents. Therefore, our findings are primarily representative of female youths, and caution should be exercised when generalizing these results to male adolescents. Future studies with larger male samples are needed to replicate and extend our findings.

### Clinical implications and future directions

Despite these limitations, the present findings have several clinical implications. First, the mediating role of neuroticism suggests that screening for this personality trait in youths experiencing depression or anxiety could help to identify those at elevated risk of NSSI, even when emotional symptoms are mild. Interventions targeting emotion regulation and impulse control, such as dialectical behavior therapy (DBT), may be particularly beneficial for youths with high levels of neuroticism. Second, the moderating effects of thyroid hormones, particularly TSH, indicate that routine thyroid function tests could offer additional biological markers for assessing NSSI risk. In particular, low TSH levels in depressed adolescents with high neuroticism may signal a higher likelihood of NSSI and require closer monitoring. Third, the distinct patterns for anxiety (fully mediated by neuroticism) versus depression (partially mediated, with thyroid modulation) suggest that management strategies should differ. Anxiety-driven NSSI may primarily respond to personality-focused interventions, whereas depression-linked NSSI may also benefit from an evaluation of HPT axis function.

Several future studies are warranted. To confirm the trend-level effects of TT3, replication in larger and more ethnically diverse samples is needed. Longitudinal designs involving repeated measurements of mood, personality, thyroid hormones and NSSI are essential for establishing temporal causality. Using collateral reports or ecological momentary assessment (EMA) could reduce recall bias in NSSI measurement. Finally, including measures of the HPA axis (e.g. cortisol) would help to determine whether other neuroendocrine systems modulate depression/anxiety-NSSI pathways, given that the HPA axis may play a different or complementary role to that of the HPT axis. In addition, given the predominantly female composition of our sample, future studies with larger and more balanced male samples are needed to confirm whether the observed mediating and moderating effects generalize to male adolescent populations.

## Conclusion

This study demonstrates that neuroticism mediates the relationship between anxious and depressed symptoms and NSSI severity in youths, with full mediation for anxiety and partial mediation for depression. Additionally, thyroid hormones modulate the depression-NSSI pathway. TT3 and FT3 positively moderate this association, while TSH negatively moderates it. TSH negatively moderates the depression-NSSI association in the full sample, and this moderating effect remains significant in the high-neuroticism subgroup after FDR correction, but not in the low-neuroticism subgroup. This indicates a neuroticism-dependent neuroendocrine mechanism. These findings integrate psychological and neuroendocrine perspectives to inform risk identification and personalized intervention strategies for NSSI in adolescents and young adults.

## Data Availability

The raw data supporting the conclusions of this article will be made available by the authors, without undue reservation.
